# Supplementary material for: A lectin receptor kinase as a potential sensor for extracellular nicotinamide adenine dinucleotide in Arabidopsis thaliana
Source: eLife. 2017 Jul 19;6:e25474. doi: 10.7554/eLife.25474 (PMC5560858; doi:10.7554/eLife.25474)
Supplement: Supplementary file 1. — (B) Receptor-like genes induced by NAD+ treatment. (C) Receptor-like kinase genes induced by P. brassicae oviposition and/or NAD+ treatment. (D) Primers used in this study. DOI: http://dx.doi.org/10.7554/eLife.25474.025 [file elife-25474-supp1.docx]

**Supplementary file 1**

**Supplementary file 1A. Well-known defense genes affected by NAD^+^ treatment**

| AGI  locus | Gene  name | NAD^+^/Water (at 4 h) | | AGI description |
| --- | --- | --- | --- | --- |
|  |  | Log_2_ (FC) | *q* Value |  |
| PTI pathway genes | | | | |
| At5g46330 | *FLS2* | 1.064 | **0.061**^*^ | Flagellin-sensitive 2 |
| At5g20480 | *EFR* | 1.177 | 0.018 | Ef-Tu receptor |
| At3g21630 | *CERK1* | 1.652 | **0.088** | Chitin elicitor receptor kinase 1 |
| At4g33430 | *BAK1* | 1.342 | 0.022 | BRI1-associated receptor kinase 1 |
| At2g39660 | *BIK1* | 1.406 | 0.044 | Botrytis-induced kinase 1 |
| At4g34390 | *XLG2* | 1.818 | 0.026 | Extra-large GTP-binding protein 2 |
| At1g51660 | *MKK4* | 2.132 | 0.033 | Mitogen-activated protein kinase kinase 4 |
| At3g21220 | *MKK5* | 1.538 | 0.025 | Mitogen-activated protein kinase kinase 5 |
| At2g19190 | *FRK1* | 5.396 | 0.012 | Flg22-induced receptor-like kinase 1 |
| At2g30250 | *WRKY25* | 1.986 | 0.018 | WRKY DNA-binding protein 25 |
| At4g23550 | *WRKY29* | 1.566 | 0.032 | WRKY DNA-binding protein 29 |
| At2g38470 | *WRKY33* | 1.08 | 0.048 | WRKY DNA-binding protein 33 |
| At5g26920 | *CBP60g* | 3.609 | 0.02 | CAM binding protein 60g |
| SA pathway genes | | | | |
| At1g64280 | *NPR1* | 1.163 | 0.048 | Nonexpressor of PR genes 1 |
| At5g45110 | *NPR3* | 2.188 | 0.023 | NPR1-like protein 3 |
| At1g02450 | *NIMIN1* | 3.667 | 0.026 | NIM-interacting 1 |
| At3g25882 | *NIMIN2* | 4.308 | 0.018 | NIM-interacting 2 |
| At1g74710 | *ICS1* | 3.416 | 0.015 | Isochorismate synthase 1 |
| At4g39030 | *EDS5* | 3.687 | 0.014 | Enhanced disease susceptibility 5 |
| At5g13320 | *PBS3* | 3.659 | 0.026 | AvrPphB susceptible 3 |
| At2g37040 | *PAL1* | 1.236 | 0.01 | Phenylalanine ammonia-lyase 1 |
| At1g73850 | *SARD1* | 3.892 | 0.015 | SAR deficient 1 |
| At3g48090 | *EDS1* | 2.996 | 0.025 | Enhanced disease susceptibility 1 |
| At3g52430 | *PAD4* | 4.234 | 0.017 | Phytoalexin deficient 4 |
| At5g14930 | *SAG101* | 1.703 | 0.025 | Senescence-associated gene 101 |
| At4g23570 | *SGT1A* | 2.851 | 0.02 | Suppressor of G2 (Two) 1a |
| At2g13810 | *ALD1* | 3.275 | 0.052 | AGD2-like defense response protein 1 |
| At1g19250 | *FMO1* | 3.89 | 0.028 | Flavin-dependent momooxygenase 1 |
| At4g12470 | *AZI1* | 2.786 | 0.042 | Azelaic acid induced 1 |
| At1g80460 | *NHO1* | 1.321 | 0.036 | Nonhost resistance 1 |
| At2g04450 | *NUDT6* | 5.848 | 0.019 | Nudix hydrolase homolog 6 |
| At4g12720 | *NUDT7* | 3.852 | 0.025 | Nudix hydrolase Homolog 7 |
| NPR1 target genes | | | | |
| At2g14610 | *PR1* | 1.272 | **0.483** | Pathogenesis-related gene 1 |
| At1g75040 | *PR5* | 1.347 | **0.182** | Pathogenesis-related gene 5 |
| At4g12010 |  | 1.026 | 0.022 | Disease resistance protein, putative |
| At5g57550 | *XTR3* | 2.195 | 0.013 | Xyloglucan endotransglycosylase 3 |
| At5g64120 |  | 2.899 | 0.02 | Peroxidase, putative |
| At1g09210 | *CRT2* | 2.46 | 0.048 | Calreticulin 2 |
| At1g30900 | *VSR6* | 3.433 | 0.02 | Vacuolar sorting receptor 6 |
| At3g54960 | *PDIL1-3* | 2.047 | 0.026 | PDI-like 1-3 |
| At4g24190 | *SHD* | 2.491 | 0.042 | Shepherd; ATP binding |
| At5g42020 | *BIP* | 3.807 | 0.029 | Luminal binding protein; ATP binding |
| At5g61790 | *CNX1* | 3.359 | 0.032 | Calnexin 1 |
| At4g31800 | *WRKY18* | 3.949 | 0.016 | WRKY DNA-binding protein 18 |
| At5g22570 | *WRKY38* | 3.539 | 0.017 | WRKY DNA-binding protein 38 |
| At4g23810 | *WRKY53* | 2.822 | 0.023 | WRKY DNA-binding protein 53 |
| At2g40750 | *WRKY54* | 1.194 | 0.048 | WRKY DNA-binding protein 54 |
| At3g01080 | *WRKY58* | 1.224 | 0.029 | WRKY DNA-binding protein 58 |
| At2g21900 | *WRKY59* | 1.703 | 0.041 | WRKY DNA-binding protein 59 |
| JA/ET pathway genes | | | | |
| At5g44420 | *PDF1.2* | -3.057 | 0.024 | Plant defensin 1.2 |
| At2g26020 | *PDF1.2b* | -1.965 | 0.025 | Plant defensin 1.2b |
| At5g44430 | *PDF1.2c* | -1.543 | 0.037 | Plant defensin 1.2c |
| At2g26010 | *PDF1.3* | -1.598 | 0.02 | Plant defensin 1.3 |

* *q* values bigger than 0.05 were marked in bold.

**Supplementary file 1B. Receptor-like genes induced by NAD^+^ treatment**

| AGI  locus | Gene  name | NAD^+^/Water  (at 4 h) | | AGI description | Homozygous T-DNA insertion lines |
| --- | --- | --- | --- | --- | --- |
|  |  | Log_2_ (FC) | *q* value |  |  |
| At4g23215 |  | 3.655 | 0.012 | Pseudogene of cysteine-rich receptor-like protein kinase family protein | SALK_105169 |
| At5g01540 | *LecRK-VI.2* | 3.596 | 0.015 | L-type lectin receptor kinase-VI.2 | SALK_070801, SAIL_796_E11, SAIL_1146_B02 |
| At5g60280 | *LecRK-I.8* | 3.239 | 0.012 | L-type lectin receptor kinase-I.8 | SALK_066416, SALK_005125, SALK_206382 |
| At2g13790 | *SERK4* | 2.368 | 0.037 | Somatic embryogenesis receptor-like kinase 4 |  |
| At2g13800 | *SERK5* | 2.075 | 0.023 | Somatic embryogenesis receptor-like kinase 5 | SALK_071634, SALK_147275 |
| At1g21250 | *WAK1* | 2.069 | **0.053*** | Cell wall-associated kinase 1 | SALK_107175, GABI_891F01 |
| At1g21240 | *WAK3* | 1.983 | 0.024 | Cell wall-associated kinase 3 | SALK_038813 |
| At4g28490 | *RLK5* | 1.937 | 0.042 | Receptor-like protein kinase 5 | SALK_021905, SALK_015074 |
| At3g45860 | *CRK4* | 1.904 | 0.036 | Cysteine-rich receptor-like kinase 4 | SALK_063969, SALK_089138 |
| At3g21900 |  | 1.739 | 0.023 | Receptor-like protein kinase-related family protein |  |
| At1g69270 | *RPK1* | 1.535 | 0.017 | Receptor-like protein kinase 1 | SALK_208360 |
| At5g01550 | *LecRK-VI.3* | 1.513 | 0.042 | L-type lectin receptor kinase-VI.3 |  |
| At5g41290 |  | 1.405 | 0.037 | Receptor-like protein kinase-related family protein |  |
| At3g08870 | *LecRK-VI.1* | 1.349 | 0.038 | L-type lectin receptor kinase VI.1 |  |
| At1g16110 | *WAKL6* | 1.313 | 0.028 | Wall associated kinase-like 6 |  |
| At1g11330 |  | 1.29 | 0.043 | S-locus lectin protein kinase family protein | SALK_143489 |
| At3g45410 | *LecRK-I.3* | 1.227 | **0.145** | L-type lectin receptor kinase I.3 | SAIL_710_D02 |
| At1g72930 | *TIR* | 1.2 | **0.203** | Toll/interleukin-1 receptor-like | SALK_204689 |
| At1g61370 |  | 1.172 | 0.033 | S-locus lectin protein kinase family protein | SALK_126675, SALK_087761 |
| At5g67280 | *RLK* | 1.053 | **0.071** | Receptor-like kinase | SALK_080358 |
| At5g46330 | *FLS2* | 1.064 | **0.061**^*^ | Flagellin-sensitive 2 |  |
| At5g20480 | *EFR* | 1.177 | 0.018 | Ef-Tu receptor |  |
| At3g21630 | *CERK1* | 1.652 | **0.088** | Chitin elicitor receptor kinase 1 |  |
| At4g33430 | *BAK1* | 1.342 | 0.022 | BRI1-associated receptor kinase 1 |  |
| At2g19190 | *FRK1* | 5.396 | 0.012 | Flg22-induced receptor-like kinase 1 |  |

* *q* values bigger than 0.05 were marked in bold.

**Supplementary file 1C. Receptor-like kinase genes induced by *P. brassicae* oviposition and/or NAD^+^ treatment**

| AGI Locus | Gene Name | *P. brassicae* 72 h^a^  log_2_ (FC) | NAD^+^ 4 h  log_2_ (FC) |
| --- | --- | --- | --- |
| At5g47850 | *CCR4* | 1.422 |  |
| At4g23200 | *CRK12* | 1.339 | 2.891 |
| At4g23220 | *CRK14* | 1.144 | 2.348 |
| At4g23190 | *CRK11*/*RLK3* | 1.417 | 2.136 |
| At4g23150 | *CRK7* | 2.63 | 2.179 |
| At3g45860 | *CRK4* | 1.17 | 1.904 |
| At4g23260 | *CRK18* | 1.275 |  |
| At4g23320 | *CRK24* | 1.09 |  |
| At4g23210 | *CRK13* | 1.401 | 4.738 |
| At4g04490 | *CRK36* | 2.42 | 3.354 |
| At4g04500 | *CRK37* | 2.278 | 3.024 |
| At4g11890 | *CRK45*/*ARCK1* | 1.993 | 5.292 |
| At2g37710 | *LecRK-IV.1* | 1.546 |  |
| At3g53810 | *LecRK-IV.2* | 1.163 |  |
| At5g01540 | *LecRK-VI.2* | 2.536 | 3.596 |
| At5g60280 | *LecRK-I.8* | 2.516 | 3.239 |
| At2g32800 | *LecRK-S.2*/*AP4.3A* | 0.864 |  |
| At1g66880 |  | 1.982 | 2.164 |
| At5g15730 | *CRLK2* | 1.176 | 1.05 |
| At1g51890 |  | 1.975 | 4.802 |
| At5g45800 | *MEE62* | 1.86 |  |
| At3g14840 | *LIK1* | 1.021 | 1.226 |
| At3g09010 |  | 1.444 | 2.227 |
| At1g16670 |  | 1.014 | 1.757 |
| At5g48380 | *BIR1* | 1.305 | 2.008 |
| At5g42440 |  | 1.029 | 2.437 |
| At1g74360 |  | 1.281 | 1.935 |
| At4g28490 | *HAESA*/*RLK5* | 1.305 | 1.937 |
| At1g09970 | *RLK7* | 1.516 | 1.628 |
| At5g25930 |  | 1.731 | 2.187 |
| At3g47090 |  | 1.084 | 1.411 |
| At1g35710 |  | 0.824 | 2.56 |
| At4g39270 |  | 1.531 | 1.751 |
| At5g58540 |  | 1.669 |  |
| At2g39660 | *BIK1* | 1.043 | 1.406 |
| At1g14370 | *APK2A*/*RBL2* | 1.454 | 2.236 |
| At4g35600 | *CX32*/*KIN4* | 1.084 | 1.42 |
| At5g47070 |  | 0.782 | 1.657 |
| At5g51770 |  | 0.949 |  |
| At1g61380 | *LORE*/*SD1-29* | 1.077 | 1.474 |
| At4g18250 |  | 2.07 | 2.643 |

^a^Data were taken from Little et al. (2007) and changed to log_2_ (FC).

**Supplementary file 1D. Primers used in this study**

| Name of the primer | Sequence (5’ to 3’) | Name of the primer | Sequence (5’ to 3’) |
| --- | --- | --- | --- |
| Identification of homozygous T-DNA insertion lines | | | |
| SALK_105169F | TTTGAACCTCGTTGAAGTTGC | SALK_105169R | TCGTCCCACAGGTTATCAAAG |
| SALK_070801F | TGAGGTCGACGAAACATATCC | SALK_070801R | AAGTGCTCATTACGTGATGGG |
| SAIL_796_E11F | CCGCAAACGATCTCTAACAAC | SAIL_796_E11R | CCTATTCAAGCCCTTTTGGAG |
| SAIL_1146_B02F | TGGTTCAACGATCAAAAATGG | SAIL_1146_B02R | GTTCGAGAATTTGTCGCAGAG |
| SALK_066416F | ACACCACAACCAACAGGTCTC | SALK_066416R | CAGAAGCTGTTTCTCAATCGG |
| SALK_206382F | AAAGGCGAGTTTCTTCTCGTC | SALK_206382R | ATGTTGTCTCCCAAAATTCCC |
| SALK_005125F | AAAGGCGAGTTTCTTCTCGTC | SALK_005125R | GCTTCCTTCTTTCCATGGTTC |
| SALK_071634F | GCAAATGAATAATCAACGTAATCC | SALK_071634R | CAAATTTGGAAGCTGAGCAAG |
| SALK_147275F | GATGAGCTCGAGAAGCATGAC | SALK_147275R | AACATTCCACTTGGTTGATGC |
| SALK_107175F  (GABI_891F01) | GCTTCTTGGTCATTCTGCTTG | SALK_107175R | TTGTGCTGACAAGATGTGACC |
| SALK_038813F | TTGGAGTTTCCTGGAAAAATC | SALK_038813R | AGAACCCGGTAACATGGAATC |
| SALK_021905F | CACCTTCCTTCTCTCCATTCC | SALK_021905R | GTTCGAGAAGTGACAAGCGAG |
| SALK_015074F | TGTTGTTACTCAATCGGACCC | SALK_015074R | TAAACCAAGATGCCACCATTC |
| SALK_063969F | ACCAAACACATGTGGGGTTAC | SALK_063969R | ATGTATGGCCAATTCTCCATG |
| SALK_089138F | GCAGACTCTACCATGCAAAGC | SALK_089138R | GTTTTGATTCCCTCCCTCAAC |
| SALK_208360F | TTGTGACATGAGACTGCGAAG | SALK_208360R | TTTTAACCGAGAGTGGTGGTG |
| SALK_143489F | TACTCCGGTGACATATAGCCG | SALK_143489R | TGCAATCATTTTTGGGAAGTG |
| SAIL_710_D02F | TGATTTGTCAACTTTTTCCCC | SAIL_710_D02R | GGTTCCTCTTCTCGGGTATTG |
| SALK_204689F | CCCCACATTTGTTATGTTTCC | SALK_204689R | AAAATTGGTCAATGCTTGCC |
| SALK_126675F | ACTCTCTTGGACTTGCATTGC | SALK_126675R | TCCAACACAGAAACTTGGTCC |
| SALK_087761F | ATACACTGGACCGAATCCACC | SALK_087761R | ACATTTCCCAAGATGTTGCAG |
| SALK_080358F | GAACTGTCACTATCTTGCGCC | SALK_080358R | CAACTCTTTCTCCGGTAACCC |
| LBa1 | TGGTTCACGTAGTGGGCCATCG | | |
| LB3 | TAGCATCTGAATTTCATAACCAATCTCGATACAC | | |
| o8409 | ATATTGACCATCATACTCATTGC | | |
| qPCR analysis | | | |
| qAt5g60280F1 | TGCAGTACCTTGACAGACAG | qAt5g60280R1 | TCATCGTCCAATTCCGTATTG |
| qAt5g60280F2 | TCATACTCTTGGTACTCCTGG | qAt5g60280R2 | GTAGACTTCTCCGAATCCTC |
| qAt5g60280F3 | TCCAGGATTGGATCTGATCTG | qAt5g60280R3 | TCTGCTCTGAAGTGTCAGTC |
| Plasmid construction | | | |
| XbaI-SalI-GFPF | GCTCTAGAGGTGTCGACATGGGTAAAGGAGAAGAAC | | |
| XhoI-GFPR | CCGCTCGAGTTATTTGTATAGTTCATCCATGC | | |
| BamHI-flLecRK-I.8F | CGGGATCCATGGCTCCAGGATTGGATCTG | | |
| SalI-flLecRK-I.8R | GCGTCGACTCGTCCAATTCCGTATTGAATC | | |
| EcoRI-ATGLecRK-I.8F | GGAATTCATGGCTCCAGGATTGGATCTG | | |
| BspHI-eLecRK-I.8R | CGCTCATGACTACTTCTGCATACTTGTTTCTTC | | |
| SacI-eLecRK-I.8F | CGAGCTCATGCAACAAGAAACTGGCTTCAGC | | |
| SalI-HisHAeLecRK-I.8R | GCGTCGACTCAGTGGTGGTGGTGGTGGTGCGCATAGTCAGGAACATCGTATGGGTAGAGAGAAGTTTTCATTTTAGGATG | | |
| EcoRI-eRLecRK-I.8F | GGAATTCCAACAAGAAACTGGCTTCAGC | | |
| SalI-eLecRK-I.8R | GCGTCGACTCAGAGAGAAGTTTTCATTTTAGGATG | | |
| EcoRI-eDORN1F | GGAATTCCAACAAGAGACAAGCTTTGTC | | |
| SalI-eDORN1R | GCGTCGACTATGTAGATACTTTCTTATGTGGAG | | |
| EcoRI-eLecRK-I.3F | GGAATTCCAACAAGAAACCGGGTTTGTC | | |
| SalI-eLecRK-I.3R | GCGTCGACTATGGAGATAATTTCTTTTTTTCCTCTTTAG | | |
| BamHI-LecRK-I.8KDF | CGGGATCCCTATACAGAAGAAACAAGTATGCAG | | |
| SalI-LecRK-I.8KDR | GCGTCGACTCATCGTCCAATTCCGTATTG | | |
| EcoRI-eLecRK-I.6F | GGAATTCCAACAAGAGACAGAGTTTATCTTTAATGG | | |
| PstI-eLecRK1.6R | AACTGCAGCTACACTGATGGTCCCTTATTTTTAGCTCTATG | | |
| XbaI-SalI-mCherryF | GCTCTAGAGGTGTCGACATGGTTTCAAAAGGTGAAGAAGATAATATGGCCATTATCAAG | | |
| XhoI-mCherryR | CCGCTCGAGCTATTTGTAGAGTTCATCCATTCCAC | | |
| SacI-AHA2F | CGAGCTCATGTCGAGTCTCGAAGATATCAAG | | |
| SalI-AHA2R | GCGTCGACCACAGTGTAGTGACTGGGAGTTTC | | |
